# Erythema and Induration of Bacillus Calmette-Guérin Scar Associated With Multisystem Inflammatory Syndrome in Children in Japan: A Case Report

**DOI:** 10.3389/fped.2022.849473

**Published:** 2022-03-11

**Authors:** Naoki Tsuboya, Hirotoshi Makino, Yoshihide Mitani, Michiko Ito, Kazunobu Ohya, Mari Morimoto, Ryo Hanaki, Noriko Yodoya, Hiroyuki Ohashi, Hirofumi Sawada, Kenji Sugiyama, Masahiro Hirayama

**Affiliations:** ^1^Department of Pediatrics, Mie University Graduate School of Medicine, Tsu, Japan; ^2^Department of Pediatrics, Mie Prefectural General Medical Center, Yokkaichi, Japan

**Keywords:** Multisystem Inflammatory Syndrome in Children (MIS-C), COVID-19, Kawasaki disease (KD), SARS-CoV-2, Bacillus Calmette-Guérin (BCG), PIMS-TS, case report

## Abstract

Multisystem Inflammatory Syndrome in Children (MIS-C) is a rare febrile disorder with multisystem organ involvement temporally associated with coronavirus 2019 infection (COVID-19) and frequently exhibits features mimicking Kawasaki disease (KD), another febrile disorder in children. The pathogenesis and the full clinical spectrum of MIS-C is poorly understood: It is still unclear whether MIS-C and KD are different syndromes or represent a common spectrum. The erythema and induration of Bacillus Calmette-Guérin (BCG) scar is one of the characteristic findings of KD, and is useful for the diagnosis in countries where BCG vaccination is mandated in infancy. Furthermore, such findings in BCG scar were also reported after SARS-CoV-2 vaccination, which may be related to molecular mimicry. However, there are no reports of changes at the BCG scar in MIS-C cases. Here, we report a case of MIS-C in a 3-year-old Hispanic boy in Japan, with erythema and induration at the BCG scar. The patient received BCG vaccination at 16 months of age in Japan. Four weeks before the onset, he had positive polymerase chain reaction (PCR) results for SARS-CoV-2 following household outbreak, although he was asymptomatic. He presented with fever and gastrointestinal symptoms, followed by the appearance of all six principal findings of complete KD. He exhibited congestive heart failure, following intravenous immunoglobulin (IVIG) therapy. He was diagnosed with MIS-C based on characteristic mucocutaneous and gastrointestinal symptoms, decreased cardiac function, and coagulopathy, in addition to laboratory data consistent with MIS-C. The BCG finding was present from the early stage of the disease. The patient was refractory to two doses of IVIGs, and the third IVIG plus prednisolone resulted in defervescence and improvement in heart failure. No coronary involvement was observed. This is the first case of erythema and induration at the BCG scar associated with MIS-C accompanied by KD features, which may give clinical and mechanistic insights in the understanding of the disease. Since the full spectrum of MIS-C is still evolving and both of them are syndromes with overlapped clinical features, further studies are warranted for deep phenotyping of MIS-C with KD features relative to KD in countries with mandatory BCG programs in infancy.

## Introduction

Multisystem Inflammatory Syndrome in Children (MIS-C) is a rare febrile disorder with multisystem organ involvement temporally associated with coronavirus 2019 infection (COVID-19), and frequently exhibits the features mimicking Kawasaki disease (KD), another febrile disorder in children (1, 2). The pathogenesis and full clinical spectrum of MIS-C are poorly understood. MIS-C and KD are currently regarded as the result of abnormal innate and adaptive immune responses characterized by an exaggerated cytokine response (3–5). Although it is still undetermined whether MIS-C and KD are different syndromes or represent a common spectrum of a disease, epidemiological, clinical, and immunological studies suggest that MIS-C is largely distinct from KD with respect to the ethnicity, the age range, the gastrointestinal and cardiovascular involvement, hematological and immunological findings, and the time interval from the exposure to SARS-CoV-2 (6, 7).

The erythema and induration of Bacillus Calmette-Guérin (BCG) scar is one of the characteristic findings of KD, and is reported to be useful for the diagnosis of this disorder in countries where BCG vaccination is mandated in infancy (8, 9). However, to the best of our knowledge, there are no reports of findings at the BCG scar in MIS-C cases.

Here, we report a case of typical MIS-C with KD features in a 3-year-old Hispanic boy accompanied by erythema and induration at the BCG scar in Japan.

## Case Description

The patient is a 3-year-old boy with no remarkable medical history, and his parents are Hispanic descendants. He was born in Japan and received the BCG vaccine at 16 months of age (885 days prior to the onset). He had no history of animal contact or traveling abroad. Four weeks prior to the onset, he was diagnosed with COVID-19 infection by polymerase chain reaction (PCR) analysis following household transmission, although he was asymptomatic. The date of the infection corresponded to the surge of SARS-CoV-2 Delta variant in the prefecture, which coincided with the fifth wave of the infection in Japan (Supplementary Figure 1) (10).

**FIGURE 1:**
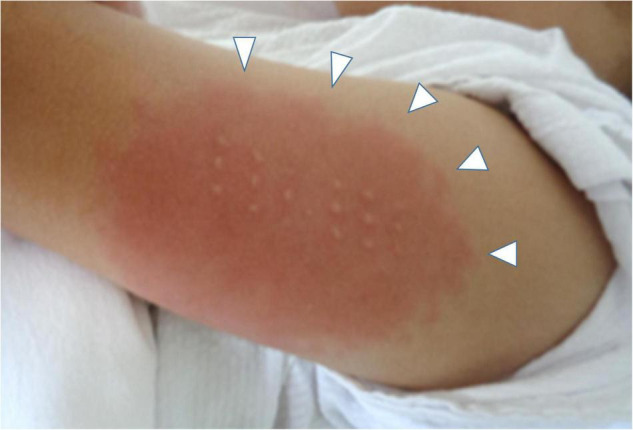
The erythema and induration at BCG scar on the day 4. White arrows indicate the skin lesion.

**FIGURE 2:**
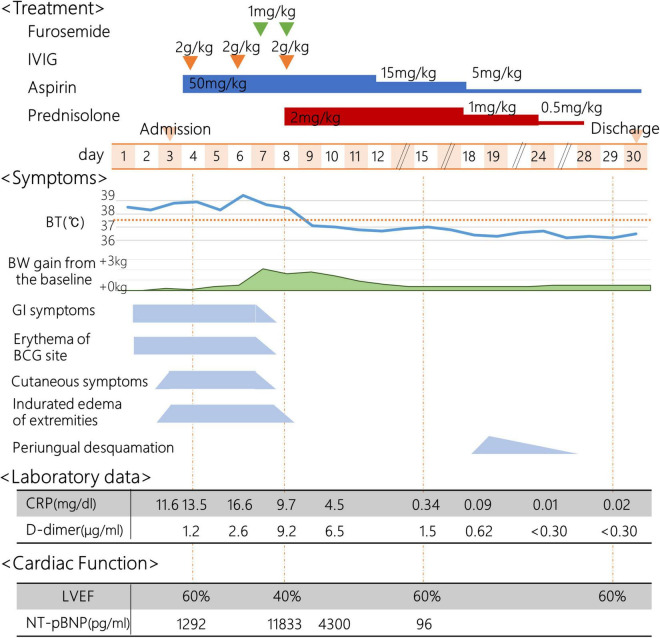
Clinical course. IVIG, intravenous immunoglobulin; BT, body temperature; BW, body weight; GI, Gastrointestinal; BCG, Bacillus Calmette-Guérin; CRP, C-reactive protein; LVEF, left ventricle ejection fraction; NT-proBNP, N-terminal pro-brain natriuretic peptide.

**FIGURE 3:**
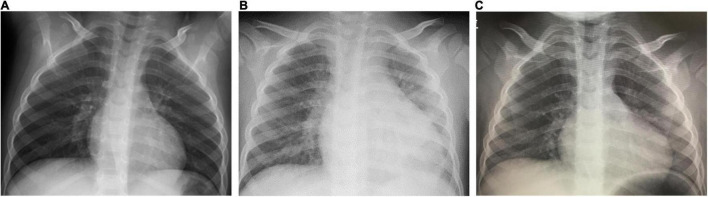
Chest X-ray findings. Chest X-ray on the day 3 [**A**, cardio thoracic ratio (CTR) 46%]; on the day 8 (**B**, CTR 62%); on the day 15 (**C**, CTR 53%).

On the day1, he developed fever of 38.5°C, and on the day 2, he presented with abdominal pain, frequent vomiting, watery diarrhea, and erythema and induration of BCG scar (Figure 1). On the day 3, his general condition became worse and he was admitted to a nearby secondary referral hospital. Physical examination revealed redness of the pharynx, abdominal tenderness, and hyperactive bowel sounds. Table 1 shows the laboratory findings. The result of SARS-CoV-2 antigen rapid test analyzed with Elecsys SARS-CoV-2 Ag^®^ (Roche Diagnostics International Ltd., Switzerland) was negative. On the day 4, he presented with bilateral bulbar conjunctival injection, cervical lymphadenopathy with tenderness, erythema of oral mucosa with cracking of lips and, rash on trunk and bilateral thighs, and erythema and firm induration of the palms and soles. He had normal heart rhythm and had no murmur. The decreases in lymphocyte count and platelet count and increases in C-reactive protein level (CRP), N-terminal pro-brain natriuretic peptide (NT-proBNP), and D-dimer were noted (Figure 2). Leukocyte count, neutrophil count, and hemoglobin level were within the reference range. The levels of aspartate aminotransferase, creatinine kinase, serum sodium, and ferritin were within the reference range (Table 1). Chest and abdominal X-ray findings were normal (Figure 3A). Echocardiography showed preserved cardiac function with a left ventricle ejection fraction (LVEF) of 60%. The patient was diagnosed with complete KD based on all six principal findings and laboratory results (8, 11). Since this patient was predicted as a good responder to intravenous immunoglobulin (IVIG) according to the risk score system (11–14), we administered IVIG (2 g/kg) and aspirin (50 mg/kg/day) on the day 4 of illness. However, the patient was resistant to the initial IVIG. After 48 h of the initial IVIG treatment, laboratory tests showed an increase in CRP level and D-dimer level compared to before initial treatment. On the day 6, a second dose of IVIG (2 g/kg) was administered as an additional treatment. The cutaneous and mucosal findings including the erythema of BCG scar gradually disappeared, although the fever persisted. Following the second IVIG administration, progressive pitting edema was observed throughout the body with a 15% weight gain from the baseline, and urine output decreased on the day 8. Chest X-ray showed cardiomegaly (62% of the cardiothoracic ratio, CTR) (Figure 3B), electrocardiogram showed an inverted T-wave at V4 induction, echocardiography showed a decreased LVEF of 40–45%, and abdominal echocardiography showed a pleural effusion. Such findings suggested cardiac failure. The patient was not in shock. The positive result of anti-SARS-CoV-2 antibody analyzed with Elecsys Anti-SARS-CoV-2 S RUO^®^ (Roche Diagnostics International Ltd., Switzerland) was confirmed.

**TABLE 1:** Time course of the laboratory findings.

Day of illness Event	Day3 Admission	Day4 Pre-treatment	Day8 Reduced cardiac function	Day15 Recovery	Reference range
Leukocyte (× 10^3^/μL)	7.8	7.1	4.9	7.4	4.5–13.5
Neutrophil (× 10^3^/μL)	5.6	5.4	1.9	2.5	1.8–8.0
Lymphocyte (× 10^3^/μL)	0.85	1.0	1.8	3.5	1.5–6.5
Hemoglobin (g/dL)	11.3	10.5	8.1	9.7	11.5–14.8
Platelets (× 10^4^/μL)	12.1	11.5	27.0	65.8	15.0–40.0
Albumin (g/dL)	3.8	3.2	1.8	3.0	3.5–5.0
Total bilirubin (mg/dL)	0.66	0.47	0.2	0.2	0.4–1.5
Aspartate aminotransferase (IU/L)	40	24	21	30	16–38
Alanine aminotransferase (IU/L)	31	22	8	20	4–25
Lactate dehydrogenase (IU/L)	278	212	184	191	286–606
Creatinine kinase (IU/L)	37	35	15	8	41–212
Blood urea nitrogen (mg/dL)	8.7	4.2	5.7	11.9	8.0–20.0
Creatinine (mg/dL)	0.29	0.25	<0.17	<0.17	0.34–0.51
Sodium (mEq/L)	131	134	139	136	138–145
Potassium (mEq/L)	3.9	3.0	3.6	3.9	3.4–4.7
Chloride (mEq/L)	96	97	104	99	98–106
Ferritin (ng/mL)	N/A	153	179	N/A	5–157
PT-international normalized ratio	1.04	0.95	1.06	0.83	0.75–1.15
Fibrinogen (mg/dL)	414	347	344	N/A	200–400
Troponin I (pg/mL)	<2.49	N/A	16.1	<0.01	<26

*N/A, not available; NT-proBNP, N-terminal pro-brain natriuretic peptide.*

On the day 8, the patient was transferred to the tertiary referral hospital. The laboratory tests showed that NT-proBNP, CRP, and D-dimer were markedly elevated, albumin level and hemoglobin level were decreased, and creatinine kinase and troponin I levels were within normal range. The throat and blood culture isolated on admission were negative. He was diagnosed with MIS-C with KD features based on the positive history of positive PCR results for SARS-CoV-2, characteristic mucocutaneous findings, gastrointestinal symptoms, decreased cardiac function, and coagulopathy, in addition to fever and laboratory data consistent with MIS-C. Other diseases were ruled out. His blood pressure could be maintained with only temporary use of diuretics. IVIG (2 g/kg) was administered in combination with prednisolone (2 mg/kg/day). The fever resolved on the next day (day 9), and the laboratory test on the day 10 showed a decrease in CRP. The edema gradually improved, and there was no recurrence of fever, skin mucous membrane symptoms, or abdominal symptoms. On the day 15, 1 week after IVIG plus prednisolone administration, laboratory data showed that the platelet count increased, and the lymphocyte count, CRP level, and NT-proBNP level were within the normal range. The enlarged cardiac silhouette disappeared (CTR 53%) on chest X-ray (Figure 3C), the inverted T-wave of V4 induction disappeared on electrocardiography, and the LVEF of 60% on echocardiography, indicating recovery of cardiac function. No coronary artery abnormalities were observed. Periungual desquamation appeared on the day 18. Prednisolone was discontinued, and the patient was discharged on the day 30 of illness. This patient was uneventful in the outpatient clinic as of at several months after the discharge.

## Discussion

This is the first case report of erythema at the BCG scar associated with typical MIS-C accompanied by KD features. The patient fulfilled the diagnostic criteria for MIS-C with persistent fever and multisystem organ involvement including mucocutaneous symptoms, gastrointestinal symptoms, reduced cardiac function, and the history of positive PCR results for SARS-CoV-2 4 weeks before in the midst of SARS-CoV2 epidemic in the local community (15, 16). Laboratory findings showed decreased lymphocyte count, platelet count, hemoglobin level, and elevated D-dimer, which were consistent with MIS-C. Hispanic ethnicity in this case may have been a risk factor for the development of MIS-C (1, 2). Although the age of the onset of MIS-C is younger than in the previous reports (2), this may be explained by the recent CDC data show that patients at 1–4 years of age account for about 20% of MIS-C (7). Other diseases were also ruled out based on behavioral history and surrounding circumstances by interview, and results of isolated bacterial cultures. Since this case however fulfilled the diagnostic criteria for KD, it is still possible that the present case could be diagnosed as KD with the history of coincidental infection of SARS-CoV2. However, the diagnosis with MIS-C is supported by the confirmed time interval from the exposure to SARS-CoV2 (positive PCR findings, exposure in the family and the epidemic in the local community), the ethnicity, the typical gastrointestinal and circulatory manifestations and laboratory findings consistent with MIS-C. Alternatively, as it is also possible that since MIS-C and KD may represent a common spectrum of a syndrome, changes in the BCG scar can be observed in such a condition. Since the full spectrum of MIS-C is still evolving and both of them are syndromes with overlapped clinical features in fact, further studies are needed on the phenotypes of MIS-C in countries with the high incidence of KD and the universal BCG program, as in Japan.

The potential reasons why there have been no case reports of BCG findings associated with MIS-C are: (i) the lower incidence of COVID-19 or MIS-C in 2020, when MIS-C began to be reported, in countries which had universal BCG vaccination programs (17–19), (ii) differences in the immunogenicity of BCG strains among countries (20), (iii) the small number of MIS-C cases in Japan, where BCG scar findings are listed in the diagnostic guidelines for KD (21), and (iv) the possibility that changes in BCG scar in MIS-C are less likely to appear in older children as in KD (9).

There are reports that erythema in the BCG scar is also associated with viral infections such as HHV6 and measles (22, 23) and influenza vaccination (24), although the mechanisms involved are not well understood. The pathogenesis of erythema in the BCG scar of KD has been suggested to include immunological mechanisms such as delayed hypersensitivity reactions associated with T cells, interleukin-1β, and tumor necrosis factor-α (25, 26), and antigen cross-reactivity between mycobacterial heat shock protein (HSP) 65 and human homolog HSP 63, which are antigenic proteins in BCG bacteria (27, 28). It is of note that several cases of BCG scar erythema after SARS-CoV-2 vaccination have been recently reported (29–31). Strong homology between the envelope protein of SARS-CoV-2 and Mycobacterium sp. antigen has been demonstrated, and specific immunity against SARS-CoV-2 is induced by BCG vaccination (32, 33). The erythema and induration of the BCG scar in MIS-C with KD features may provide an insight into the mechanism of the syndrome. It is possible that the changes of the BCG scar in MIS-C could be specifically related to the Japanese strain of BCG. It is reported that there are differences in antigenicity and immune response depending on the strain of BCG vaccination (20). Previous studies showed that early Japanese strain of BCG has higher bacterial counts than the late strains and may produce a stronger immune response (34, 35). In KD, there is a high frequency of positive BCG findings in Japan using the Japanese strain (9). In contrast, reports from India, Russia, and Latin America using non-Japanese strains show only sporadic cases of KD with findings at BCG inoculation site (36–38). This case is unique in that a young Hispanic child, which is a risk to develop MIS-C, was vaccinated with the Japanese BCG strain. Since erythema at BCG scar in KD frequently appears within 1–2 years after vaccination (9, 39), it is possible that BCG findings are more likely to be observed in infants with MIS-C as well through any immunological mechanisms shared by both disorders.

## Conclusion

We reported a case of a 3-year-old Hispanic boy who received BCG vaccination in Japan, diagnosed as MIS-C with KD features accompanied by erythema of the BCG scar. Since the history of COVID-19 may be missed in small children with mild symptoms, BCG scar changes, which was observed as early as on the day 2 in the present case, would be an alert to clinicians caring febrile children suspected of MIS-C as well as KD in countries with universal BCG program in infancy. The present case warrants the further phenotyping of MIS-C with KD features relative to KD with respect to BCG scar changes in such countries and may give an insight into the mechanisms of MIS-C.

## Data Availability Statement

The raw data supporting the conclusions of this article will be made available by the authors, without undue reservation.

## Ethics Statement

Written informed consent was obtained from the individual(s), and minor(s)’ legal guardian/next of kin, for the publication of any potentially identifiable images or data included in this article.

## Author Contributions

NT, HM, YM, MI, KO, NY, HO, HS, KS, and MH managed the patient, contributed to the conception of the study, and drafted the manuscript. YM and MH critically reviewed the manuscript. All authors read and approved the final manuscript.

## Conflict of Interest

The authors declare that the research was conducted in the absence of any commercial or financial relationships that could be construed as a potential conflict of interest.

## Publisher’s Note

All claims expressed in this article are solely those of the authors and do not necessarily represent those of their affiliated organizations, or those of the publisher, the editors and the reviewers. Any product that may be evaluated in this article, or claim that may be made by its manufacturer, is not guaranteed or endorsed by the publisher.
