# Supplementary figures and images for: Erythema and Induration of Bacillus Calmette-Guérin Scar Associated With Multisystem Inflammatory Syndrome in Children in Japan: A Case Report
Source: Front Pediatr. 2022 Mar 11;10:849473. doi: 10.3389/fped.2022.849473 (PMC8963203; doi:10.3389/fped.2022.849473)

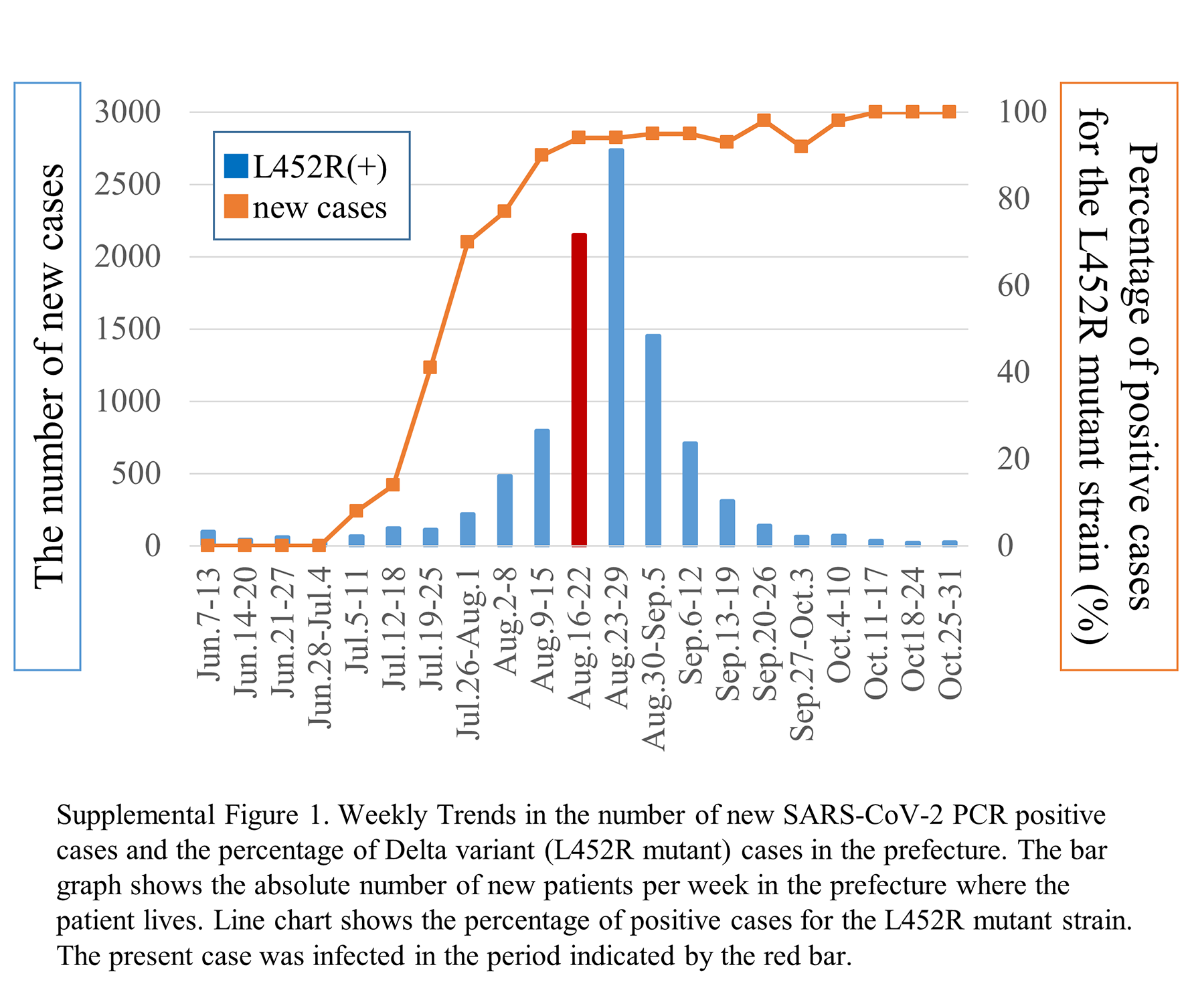

Supplement: Supplementary file 1 [file Image_1.TIF]
